# Bioactive Constituents from the Aerial Parts of *Pluchea indica* Less

**DOI:** 10.3390/molecules23092104

**Published:** 2018-08-21

**Authors:** Jingya Ruan, Zheng Li, Jiejing Yan, Peijian Huang, Haiyang Yu, Lifeng Han, Yi Zhang, Tao Wang

**Affiliations:** 1 Tianjin State Key Laboratory of Modern Chinese Medicine, Tianjin University of Traditional Chinese Medicine, 312 Anshanxi Road, Nankai District, Tianjin 300193, China; Ruanjy19930919@163.com (J.R.); wo15510977612@163.com (Z.L.); 17320072093@163.com (J.Y.); 2 Tianjin Key Laboratory of TCM Chemistry and Analysis, Institute of Traditional Chinese Medicine, Tianjin University of Traditional Chinese Medicine, 312 Anshanxi Road, Nankai District, Tianjin 300193, China; hpjforever@sina.com (P.H.); hyyu@tjutcm.edu.cn (H.Y.); hanlifeng_1@sohu.com (L.H.)

**Keywords:** *Pluchea indica* Less., chemical compositions, RAW 264.7 cells, anti-inflammatory activities

## Abstract

Four new thiophenes, (3′′*R*)-pluthiophenol (**1**), (3′′*R*)-pluthiophenol-4′′-acetate (**2**), 3′′-ethoxy-(3′′*S*)-pluthiophenol (**3**), 3′′-ethoxy-(3′′*S*)-pluthiophenol-4′′-acetate (**4**), together with twenty-five known compounds were obtained from the 70% ethanol-water extract of the aerial parts of *Pluchea indica* Less. Their structures were elucidated by spectroscopic methods. Among the known isolates, compounds **7**, **8**, **11**, **14**, **15**, **18**, **20**, **23**, **25**–**27** were isolated from Asteraceae family firstly, while compounds **6**, **9**, **10**, **12**, **13**, **16**, **19**, **21**, **28** were isolated from *Pluchea* genus for the first time. Meanwhile, compounds **1**, **2**, **10**, **13**, **18**, **23** displayed significant inhibitory activities on LPS-induced NO production at 40 µM from RAW 264.7 macrophages, while compounds **3**, **4**, **26**–**29** possessed moderate inhibitory effects.

## 1. Introduction

As one of the largest families, the Asteraceae (Compositae) family contains about 1600–1700 genera and 24,000–30,000 species. Most of the Asteraceae family plants are herbs and shrubs, and have been widely used as herbal medicines since ancient times all over the world [1]. *Pluchea indica* Less., belongs to *Pluchea* genus, Asteraceae family, is a 1 to 2 meters high shrub. It mainly distributes in the tropical and subtropical regions of Africa, Asia, America, Australia, and China’s southern provinces. As an amphibious woody semi-mangrove plant, it plays an important role in maintaining the ecological balance in the coastal areas of Southeast Asia in China [2]. As a folk medicine in Guangxi, it exhibits the function of softening hardness and dissolving lump [3]. As a type of food, it possesses the activity of warming the stomach [4]. Its main chemical compositions are thiophenes, quinic acids, sesquiterpenes, lignans, flavonoids, and sterols [2]. Pharmacological studies have shown that the plant exhibits many pharmacological functions such as anti-inflammatory [5], anti-cancer [6], anti-oxidant [7], anti-microbial [8], and insecticidal activities [9].

Through the summary of relevant literature, it is found that the pharmacodynamic material basis is not yet clear for the lack of systematic research on the plant. In the course of studying the anti-inflammatory activity of various medicinal plants, 70% EtOH extract of *P. indica* was found to possess significant in vitro anti-inflammatory bioactivity. Based on the anti-inflammatory activity on LPS-induced NO production from RAW 264.7 macrophages, a systematic chemical component study of *P. indica* aerial parts was carried out. In this paper, the isolation and identification of constituents were described as well as their inhibitory effect on the production of NO in RAW 264.7 cells induced by LPS.

## 2. Results and Discussion

**Figure 1:**
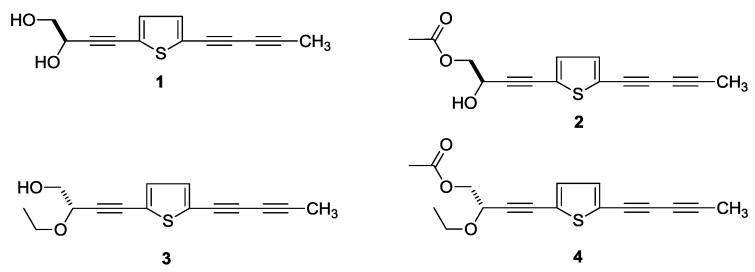
The new compounds **1**–**4** obtained from the aerial parts of *P. indica*.

**Figure 2:**
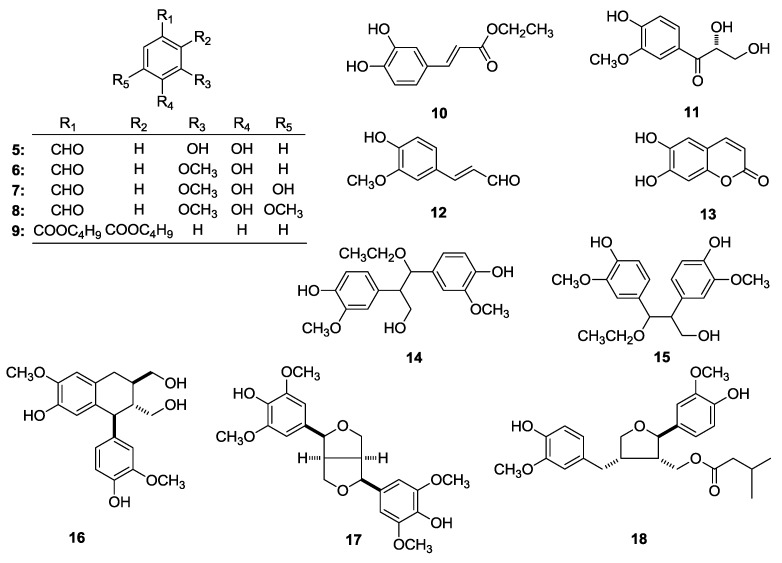

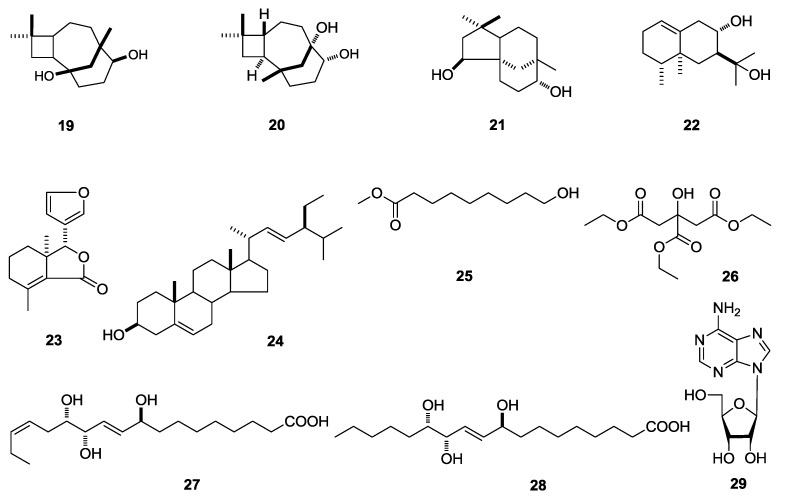
The known compounds **5**–**29** obtained from the aerial parts of *P. indica*.

**Figure 3:**
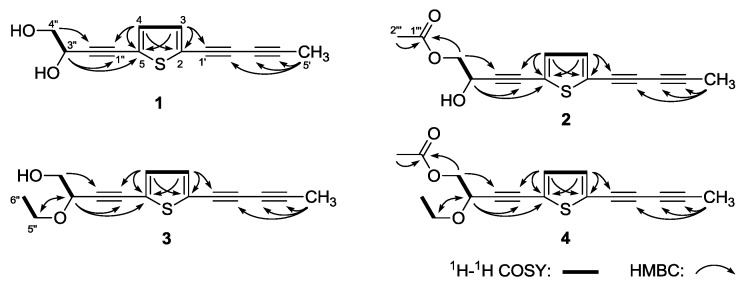
The main ^1^H-^1^H COSY and HMBC correlations of **1**–**4**.

In the course of our investigation of the bioactive constituents from the 70% ethanol-water (EtOH) extract of the aerial parts of *P. indica*, four new thiophenes, named as (3′′*R*)-pluthiophenol (**1**), (3′′*R*)-pluthiophenol-4′′-acetate (**2**), 3′′-ethoxy-(3′′*S*)-pluthiophenol (**3**), 3′′-ethoxy-(3′′*S*)-pluthiophenol-4′′-acetate (**4**) (Figure 1) as well as twenty-five known compounds, 3,4-dihydroxy benzaldehyde (**5**) [10], vanillin (**6**) [11], 3,4-dihydroxy-5-methoxybenzaldehyde (**7**) [12], syringicaldehyde (**8**) [13], dibutylphthalate (**9**) [14], ethyl caffeate (**10**) [15], 2,3-dihydroxy-1-(4-hydroxy-3-methoxyphenyl)-propan-1-one (**11**) [16], *trans*-coniferyl aldehyde (**12**) [17], esculetin (**13**) [18], *threo*-2,3-bis(4-hydroxy-3-methoxyphenyl)-3-ethoxypropan-1-ol (**14**) [19], *erythro*-2,3-bis(4-hydroxy-3-methoxyphenyl)-3-ethoxypropan-1-ol (**15**) [20], (+)-isolariciresinol (**16**) [16,21], (–)-(7*S*,7′*S*,8*R*,8′*R*)-4,4′-dihydroxy-3,3′,5,5′-pentamethoxy-7,9′:7′,9-diepoxylignane (**17**) [22,23], (+)-9′-isovaleryllariciresinol (**18**) [24,25,26], caryolane-1,9β-diol (**19**) [27], (8*R*,9*R*)-isocaryolane-8,9-diol (**20**) [28], clovane-2α,9β-diol (**21**) [27], valenc-1(10)-ene-8,11-diol (**22**) [2], fraxinellone (**23**) [29], stigmasterol (**24**) [30], methyl 9-hydroxynonanoate (**25**) [31], triethyl citrate (**26**) [32], 9,12,13-trihydroxyoctadeca-10(*E*),15(*Z*)-dienoic acid (**27**) [33], pinellic acid (**28**) [34], adenosine (**29**) [35] (Figure 2) were obtained.

(3′′*R*)-Pluthiophenol (**1**) was isolated as yellow oil. Its molecular formula was revealed to be C_13_H_10_O_2_S by positive ESI-Q-Orbitrap MS analysis (*m*/*z* 231.04726 [M + H]^+^, calcd for C_13_H_11_O_2_S, 231.04743). The characteristic absorptions in its IR spectrum suggested the presences of hydroxyl (3312 cm^−1^), thiophene ring (3105, 1448 cm^−1^), and alkynyl (2222 cm^−1^). Its ^1^H-NMR (CD_3_OD, 500 MHz) (Table 1) spectrum indicated the presence of one methyl [δ 2.02 (3H, s, H_3_-5′)], one hydroxymethyl [δ 3.64 (1H, dd, *J* = 7.0, 11.5 Hz), 3.68 (1H, dd, *J* = 5.0, 11.5 Hz), H_2_-4′′], one oxygenated methine [δ 4.55 (1H, dd, *J* = 5.0, 7.0 Hz, H-3′′)], and a couple of olefinic protons [δ 7.08 (1H, d, *J* = 4.0 Hz, H-4), 7.15 (1H, d, *J* = 4.0 Hz, H-3)]. The four carbon signals [δ 124.6 (C-2), 125.9 (C-5), 133.3 (C-4), 134.9 (C-3)] in the low field area of ^13^C-NMR (CD_3_OD, 125 MHz) spectrum, combining with the special coupling constant (*J*_H-3,4_ = 4.0 Hz) and MS data confirmed the existence of the thiophene ring. The ^1^H-^1^H COSY spectrum of **1** indicated the presence of two partial structures written in bold lines as shown in Figure 3. Furthermore, in the HMBC experiment, the long-range correlations were observed from δ_H_ 7.15 (H-3) to δ_C_ 66.8 (C-1′), 124.9 (C-2), 125.9 (C-5); δ_H_ 7.08 (H-4) to δ_C_ 78.1 (C-1′′), 124.9 (C-2), 125.9 (C-5); δ_H_ 2.02 (H_3_-5′) to δ_C_ 64.6 (C-3′), 80.1 (C-2′), 84.5 (C-4′); δ_H_ 4.55 (H-3′′) to δ_C_ 78.1 (C-1′′), 94.5 (C-2′′), 125.9 (C-5); δ_H_ 3.64, 3.68 (H_2_-4′′) to δ_C_ 64.6 (C-3′′), 94.5 (C-2′′). Consequently, the planar structure of **1** was determined. Finally, through the comparison of the optical rotation {${\left[\alpha \right]}_{D}^{25}$ + 11.4° (MeOH)} of **1** with those of (*R*)- and (*S*)-(3*E*)-2-hydroxy-2-methyl-4-[1,8:4,5-bis(methylenedioxy)-2-naphthyl]but-3-enyl acetate, {*R*: ${\left[\alpha \right]}_{D}^{20}$ + 22.6° (MeOH); *S*: ${\left[\alpha \right]}_{D}^{20}$ − 20.0° (MeOH)], respectively} [36], its absolute configuration was elucidated to be 3′′*R*.

(3′′*R*)-Pluthiophenol-4′′-acetate (**2**) was obtained as yellow oil with positive optical rotation ${\left[\alpha \right]}_{D}^{25}$ + 7.3° (MeOH)}. The molecular formula, C_15_H_12_O_3_S of **2** was determined from ESI-Q-Orbitrap MS (*m*/*z* 273.05781 [M + H]^+^, calcd for C_15_H_13_O_3_S, 273.05799) analysis, which was 42 Da more than that of **1**, suggesting that there was one more acetyl group in **2**. Meanwhile, the ^1^H-, ^13^C- (Table 2, CD_3_OD) and 2D- (^1^H-^1^H COSY, HSQC) NMR spectra verified the existence of the acetyl group [δ_H_ 2.08 (3H, s, H_3_-2′′′), δ_C_ 172.5 (C-1′′′)]. The acetyl group was elucidated to substitute in C-4′′ by the long-range correlations observed from H-4′′ to C-1′′′ in the HMBC experiment. Similarly, according to the optical rotation, the absolute configuration of **2** was determined to be 3′′*R* [36], and its structure was determined to be (3′′*R*)-pluthiophenol-4′′-acetate. 

The planar structures of **1** and **2** had been reported by Bitew et al. [37], while their absolute configurations were not being determined. Here, they were clarified by the comparison of optical rotation with those of known compounds [36] for the first time.

3′′-Ethoxy-(3′′*S*)-pluthiophenol (**3**), yellow oil, the molecular formula, C_15_H_14_O_2_S (*m*/*z* 259.07875 [M + H]^+^, calcd for C_15_H_15_O_2_S, 259.07873) was determined by ESI-Q-Orbitrap MS. Except for the similar aglycone with **1** indicated by its ^1^H- and ^13^C-NMR (Table 3) spectra, there was one more ethoxy signal [δ 1.24 (3H, t like, *ca. J* = 7 Hz, H_3_-6′′), 3.55, 3.83 (1H each, both dq, *J* = 7.0, 9.0 Hz, H_2_-5′′)] in **3**. The ethoxy was clarified to link to C-3′′ position by the long-range correlation observed from δ_H_ 3.55, 3.83 (H_2_-5′′) to δ_C_ 15.5 (C-6′′), 72.7 (C-3′′). At last, its absolute configuration was elucidated to be 3′′*S* by the optical rotation {${\left[\alpha \right]}_{D}^{25}$ − 16.7° (MeOH)} determination [36].

3′′-Ethoxy-(3′′*S*)-pluthiophenol-4′′-acetate (**4**) was isolated as yellow oil. The ESI-Q-Orbitrap MS {*m*/*z* 301.08969 [M + H]^+^ (calcd for C_17_H_17_O_3_S, 301.08929)} and ^1^H-, ^13^C- (Table 4, CD_3_OD), 2D- (^1^H-^1^H COSY, HSQC, HMBC) NMR experiments suggested that there was one more acetyl group [δ_H_ 2.07 (3H, s, H_3_-2′′′), δ_C_ 172.3 (C-1′′′)] at C-4′′ of aglycone than **3**. Finally, comparing the optical rotation {${\left[\alpha \right]}_{D}^{25}$ − 8.9° (MeOH)} with reference [36], the absolute configuration of **4** was revealed to be 3′′*S*. Thus, its structure was determined as 3′′-ethoxy-(3′′*S*)-pluthiophenol-4′′-acetate.

The structures of known compounds **5**–**29** were identified by comparing their ^1^H-, ^13^C-NMR data with references.

The potential *in vitro* anti-inflammatory effects of 70% EtOH extract (PI) and 95% EtOH eluent (PIE) and compounds **1**–**29** obtained from the aerial parts of *P. indica* on LPS-stimulated NO production were accomplished by pretreating RAW 264.7 macrophages cells with them for 1 h before stimulating with LPS (500 ng/mL) for 24 h, respectively. Griess reagent (St. Louise, MO, USA) was used to measure NO concentrations in the culture medium. Comparing to unstimulated normal (negative control), NO production in LPS-stimulated RAW 264.7 macrophages was markedly induced (Table 5). PI and PIE displayed potential inhibitory activities on LPS-induced NO production at 100 µg/mL. Further, using the same activity screening assay, the compounds isolated from active fractions were tested at a final concentration of 40 µM. Under this concentration, cells showed no significant influence on cell viability by dimethyl thiazolyl diphenyl tetrazolium (MTT) assay. Compared with untreated cells, the changes in cell viability were less than 10% (data not shown). As results, compounds **1**, **2**, **10**, **13**, **18**, **23** showed significant inhibitory effects at 40 µM, while **3**, **4**, **26**–**29** possessed moderate in vitro anti-inflammatory activity. These results suggested that compounds **1**, **2**, **10**, **13**, **18**, **23** may exhibit potent anti-inflammatory activity.

## 3. Experimental

### 3.1. General

NMR spectra were tested on a Bruker 500 MR NMR spectrometer (Bruker BioSpin AG Industriestrasse 26 CH-8117, Fällanden, Switzerland) at 500 MHz for ^1^H- and 125 MHz for ^13^C-NMR (internal standard: TMS). Positive and negative -ion HRESI-TOF/Orbitrap-MS were determined on Thermo UHPLC-ESI-Q-Orbitrap MS spectrometer (Thermo, Waltham, MA, USA) and Agilent Technologies 6520 Accurate-Mass Q-Tof LC/MS spectrometer (Agilent Corp., Santa Clara, CA, USA). Optical rotations, UV and IR spectra were run on a Rudolph Autopol^®^ IV automatic polarimeter (l = 50 mm) (Rudolph Research Analytical, Hackettstown NJ, USA), Varian Cary 50 UV-Vis (Varian, Inc., Hubbardsdon, MA, USA) and Varian 640-IR FT-IR spectrophotometer (Varian Australia Pty Ltd., Mulgrave, Australia), respectively.

CC were performed on macroporous resin D101 (Haiguang Chemical Co., Ltd., Tianjin, China), Silica gel (48–75 μm, Qingdao Haiyang Chemical Co., Ltd., Qingdao, China), ODS (50 μm, YMC Co., Ltd., Tokyo, Japan), and Sephadex LH-20 (Ge Healthcare Bio-Sciences, Uppsala, Sweden). Preparative high-performance liquid chromatography (Prep-HPLC) column: Cosmosil 5C_18_-MS-II (4.6 mm × 250 mm) and (20 mm i.d. × 250 mm, Nakalai Tesque, Inc., Tokyo, Japan); Wacopak Navi C_30_-5 (4.6 mm × 250 mm) and (7.5 mm × 250 mm, Wako Pure Chemical Industries) were used to separate the constituents.

### 3.2. Plant Material

The aerial parts *of Pluchea indica* Less. were collected from Hepu city, Guangxi province, China and identified by Dr. Wei Songji (Zhuang Medical College, Guanxi University of Chinese Medicine). The voucher specimen was deposited at the Academy of traditional Chinese Medicine of Tianjin University of TCM.

### 3.3. Extraction and Isolation

The dried aerial parts of *P. indica* (10.0 kg) were refluxed three times with 70% EtOH. A 70% EtOH extract (1851.0 g) was provided by evaporating the solvent under pressure. Dissolved the residue in H_2_O, and the residue was then subjected to D101 CC (H_2_O → 95% EtOH), H_2_O (1110.2 g) and 95% EtOH (224.7 g) eluent were afforded, respectively.

The 95% EtOH eluent (160.7 g) was subjected to silica gel CC [CHCl_3_-MeOH (100:1 → 100:5, *v*/*v*) → CHCl_3_-MeOH-H_2_O (10:3:1 → 7:3:1 → 6:4:1 → 5:5:1, *v/v/v*, lower layer) → MeOH] to yield nine fractions (Fraction 1–Fraction 9).

Fraction 2 (0.6 g) was separated by silica gel CC [Hexane → Hexane-EtOAc (25:1 → 100:7 → 10:1, *v*/*v*) → EtOAc], and eight fractions (Fraction 2-1–Fraction 2-8) were obtained. Fraction 2-4 (89.2 mg) was purified by pHPLC [CH_3_CN-H_2_O (73:27, *v*/*v*) + 1% HAc, Cosmosil 5C_18_-MS-II column] to yield 3′′-ethoxy-(3′′*S*)-pluthiophenol-4′′-acetate (**4**, 22.8 mg) and fraxinellone (**23**, 3.5 mg).

Fraction 3 (4.2 g) was subjected to SiO_2_ gel CC [Hexane → Hexane-EtOAc (100:1 → 100:3 → 25:1 → 20:1 → 100:7 → 10:1 → 5:1, *v*/*v*) → EtOAc], eleven fractions (Frction 3-1–Fraction 3-11) were obtained. Fraction 3-4 (219.6 mg) was separated by pHPLC [CH_3_CN-H_2_O (32:68, *v*/*v*) + 1% HAc, Cosmosil 5C_18_-MS-II column] to afford stigmasterol (**24**, 27.7 mg). Fraction 3-5 (253.9 mg) was isolated by pHPLC [MeOH-H_2_O (85:15, *v*/*v*) + 1% HAc, Cosmosil 5C_18_-MS-II column] to yield (3′′*R*)-pluthiophenol-4′′-acetate (**2**, 12.3 mg) and dibutylphthalate (**9**, 18.1 mg). Fraction 3-6 (139.7 mg) was purified by pHPLC [CH_3_CN-H_2_O (20:80, *v*/*v*) + 1% HAc, Cosmosil 5C_18_-MS-II column] to obtain vanillin (**6**, 7.4 mg). Fraction 3-7 (195.8 mg) was separated by pHPLC [CH_3_CN-H_2_O (23:77, *v*/*v*) + 1% HAc, Cosmosil 5C_18_-MS-II column] to afford triethyl citrate (**26**, 9.6 mg). Fraction 3-8 (342.9 mg) was isolated by pHPLC [CH_3_CN-H_2_O (23:77, *v*/*v*) + 1% HAc, Cosmosil 5C_18_-MS-II column] to obtain *trans*-coniferyl aldehyde (**12**, 12.3 mg). Fraction 3-10 (133.8 mg) was purified by pHPLC [CH_3_CN-H_2_O (50:50, *v*/*v*) + 1% HAc, Cosmosil 5C_18_-MS-II column] to yield syringicaldehyde (**8**, 7.4 mg). 

Fraction 4 (5.3 g) was isolated by ODS CC [MeOH-H_2_O (30% → 40% → 50% → 58% → 60% → 70% → 80% → 100%, *v*/*v*)] to afford thirteen fractions (Fraction 4-1–Fraction 4-13). Fraction 4-5 (244.9 mg) was subjected to pHPLC [MeOH-H_2_O (43:57, *v*/*v*) + 1% HAc, Cosmosil 5C_18_-MS-II column], eight fractions (Fraction 4-5-1–Fraction 4-5-8) were obtained. Fraction 4-5-4 (40.6 mg) was further separated by pHPLC [CH_3_CN-H_2_O (25:75, *v*/*v*) + 1% HAc, Cosmosil 5C_18_-MS-II column] to afford seven fractions (Fraction 4-5-4-1–Fraction 4-5-4-7). Among them, Fraction 4-5-4-5 (11.9 mg) was identified as (–)-(7*S*,7′*S*,8*R*,8′*R*)-4,4′-dihydroxy-3,3′,5,5′-pentamethoxy-7,9′:7′,9-diepoxylignane (**17**, 11.9 mg). Fraction 4-5-4-2 (6.1 mg) was purified by [CHCl_3_-MeOH (100:3, *v*/*v*) → MeOH] to yield *threo*-2,3-bis(4-hydroxy-3-methoxyphenyl)-3-ethoxypropan-1-ol (**14**, 2.5 mg) and *erythro*-2,3-bis(4-hydroxy-3-methoxyphenyl)-3-ethoxypropan-1-ol (**15**, 2.1 mg). Fraction 4-6 (405.9 mg) was isolated by pHPLC [CH_3_CN-H_2_O (25:75, *v*/*v*) + 1% HAc, Cosmosil 5C_18_-MS-II column] to obtain ethyl caffeate (**10**, 41.5 mg). Fraction 4-10 (536.4 mg) was purified by pHPLC [CH_3_CN-H_2_O (41:59, *v*/*v*) + 1% HAc, Cosmosil 5C_18_-MS-II column] to yield (3′′*R*)-pluthiophenol (**1**, 69.6 mg) and caryolane-1,9β-diol (**19**, 13.3 mg). Fraction 4-11 (414.0 mg) was subjected to pHPLC [CH_3_CN-H_2_O (41:59, *v*/*v*) + 1% HAc, Cosmosil 5C_18_-MS-II column], six fractions were obtained (Fraction 4-11-1–Fraction 4-11-6). Among them, fractions 4-11-3 and 4-11-4 were elucidated as (8*R*,9*R*)-isocaryolane-8,9-diol (**20**, 16.7 mg) and (+)-9′-isovaleryllariciresinol (**18**, 14.2 mg), respectively. Fraction 4-11-2 (14.7 mg) was purified by pHPLC [MeOH-H_2_O (75:25, *v*/*v*) + 1% HAc, Cosmosil 5C_18_-MS-II column] to afford clovane-2α,9β-diol (**21**, 4.8 mg). Fraction 4-11-5 (66.3 mg) was further isolated by pHPLC [MeOH-H_2_O (65:35, *v*/*v*) + 1% HAc, Wacopak Navi C_30_-5 column], and 3′′-ethoxy-(3′′*S*)-pluthiophenol (**3**, 9.3 mg) was yield. Fraction 4-12 (314.8 mg) was separated by pHPLC [CH_3_CN-H_2_O (38:62, *v*/*v*) + 1% HAc, Cosmosil 5C_18_-MS-II column] to obtain valenc-1(10)-ene-8,11-diol (**22**, 19.8 mg).

Fraction 5 (8.0 g) was separated by Sephadex LH-20 CC [CHCl_3_-MeOH (1:1, *v*/*v*)] to afford four fractions (Fraction 5-1–Fraction 5-4). Fraction 5-2 (3.3 g) was isolated by ODS CC [MeOH-H_2_O (30% → 42% → 57% → 100%, *v*/*v*)], and ten fractions (Fraction 5-2-1–Fraction 5-2-10) were yielded. Fraction 5-2-1 (394.5 mg) was isolated by pHPLC [MeOH-H_2_O (23:77, *v*/*v*) + 1% HAc, Cosmosil 5C_18_-MS-II column] to afford 3,4-dihydroxy benzaldehyde (**5**, 44.2 mg), 3,4-dihydroxy-5-methoxybenzaldehyde (**7**, 9.1 mg), 2,3-dihydroxy-1-(4-hydroxy-3-methoxyphenyl)-propan-1-one (**11**, 6.6 mg), and esculetin (**13**, 12.0 mg). Fraction 5-2-8 (207.7 mg) was subjected to pHPLC [CH_3_CN-H_2_O (40:60, *v*/*v*) + 1% HAc, Cosmosil 5C_18_-MS-II column], methyl 9-hydroxynonanoate (**25**, 6.9 mg) was obtained. Fraction 5-2-9 (143.3 mg) was further purified by pHPLC [CH_3_CN-H_2_O (41:59, *v*/*v*) + 1% HAc, Cosmosil 5C_18_-MS-II column] to yield (3′′*R*)-pluthiophenol (**1**, 5.5 mg), 9,12,13-trihydroxyoctadeca-10(*E*),15(*Z*)-dienoic acid (**27**, 14.1 mg) and pinellic acid (**28**, 3.6 mg).

Fraction 7 (46.1 g) was isolated by Sephadex LH-20 CC [CHCl_3_-MeOH (1:1, *v*/*v*)] to obtained three fractions (Fraction 7-1–Fraction 7-3). Fraction 7-2 (15.5 g) was further separated by ODS CC [MeOH-H_2_O (30% → 40% → 50% → 60% → 70% → 100%, *v*/*v*)], and ten fractions (Fraction 7-2-1–Fraction 7-2-10) were given. Fraction 7-2-3 (1500.0 mg) was subjected to pHPLC [CH_3_CN-H_2_O (18:82, *v*/*v*) + 1% HAc, Cosmosil 5C_18_-MS-II column] to obtain eleven fractions (Fraction 7-2-3-1–Fraction 7-2-3-11). Fraction 7-2-3-1 (156.2 mg) was purified by pHPLC [MeOH-H_2_O (15:85, *v*/*v*) + 1% HAc, Cosmosil 5C_18_-MS-II column] to yield adenosine (**29**, 5.3 mg). Fraction 7-2-3-7 (178.3 mg) was further isolated by pHPLC [CH_3_CN-H_2_O (20:80, *v*/*v*) + 1% HAc, Cosmosil 5C_18_-MS-II column] to obtain (+)-isolariciresinol (**16**, 7.4 mg).

*(3′′R)-Pluthiophenol* (**1**): Yellow oil; ${\left[\alpha \right]}_{D}^{25}$ + 11.4° (c = 0.04, MeOH); UV *λ*_max_ (MeOH) nm (log *ε*): 209 (4.45), 235 (3.92), 246 (4.07), 319 (4.49), 340 (4.47); IR *ν*_max_ (KBr) cm^−1^: 3312, 3105, 2955, 2919, 2871, 2467, 2222, 2148, 1448, 1077, 1022, 804; ^1^H- and ^13^C-NMR data, see Table 1; ESI-Q-Orbitrap MS: Positive-ion mode *m*/*z* 231.04726 [M + H]^+^ (calcd for C_13_H_11_O_2_S, 231.04743).

*(3′′R)-Pluthiophenol-4′′-acetate* (**2**): Yellow oil; ${\left[\alpha \right]}_{D}^{25}$ + 7.3° (c = 0.06, MeOH); UV *λ*_max_ (MeOH) nm (log *ε*): 208 (4.54), 235 (4.01), 246 (4.16), 319 (4.58), 340 (4.56); IR *ν*_max_ (KBr) cm^−1^: 3099, 2977, 2233, 1745, 1520, 1448, 1381, 1326, 1229, 1106, 1046, 807; ^1^H- and ^13^C-NMR data, see Table 2; ESI-Q-Orbitrap MS: Positive-ion mode *m*/*z* 273.05781 [M + H]^+^ (calcd for C_15_H_13_O_3_S, 273.05799).

*3′′-Ethoxy-(3′′S)-pluthiophenol* (**3**): Yellow oil; ${\left[\alpha \right]}_{D}^{25}$ – 16.7° (c = 0.06, MeOH); UV *λ*_max_ (MeOH) nm (log *ε*): 208 (4.47), 235 (3.99), 246 (4.11), 319 (4.50), 340 (4.47); IR *ν*_max_ (KBr) cm^–1^: 3439, 3097, 2975, 2931, 2876, 2231, 1447, 1376, 1327, 1118, 807; ^1^H- and ^13^C-NMR data, see Table 3; ESI-Q-Orbitrap MS: Positive-ion mode *m*/*z* 259.07875 [M + H]^+^ (calcd for C_15_H_15_O_2_S, 259.07873).

*3′′-Ethoxy-(3′′S)-pluthiophenol-4′′-acetate* (**4**): Yellow oil; ${\left[\alpha \right]}_{D}^{25}$ – 8.9° (c = 0.04, MeOH); UV *λ*_max_ (MeOH) nm (log *ε*): 208 (4.72), 235 (4.18), 246 (4.34), 319 (4.76), 340 (4.73); IR *ν*_max_ (KBr) cm^−1^: 3098, 2976, 2228, 1745, 1445, 1377, 1330, 1231, 1105, 1048, 808; ^1^H- and ^13^C-NMR data, see Table 4; ESI-Q-Orbitrap MS: Positive-ion mode *m*/*z* 301.08969 [M + H]^+^ (calcd for C_17_H_17_O_3_S, 301.08929).

### 3.4. In Vitro Anti-Inflammatory Assay

#### 3.4.1. Materials 

Lipopolysaccharides (LPS) and dexamethasone (Dex) were purchased from Sigma Chemical (St. Louise, MO, USA); penicillin and streptomycin were purchased from Thermo Fisher Scientific (Waltham, MA, USA); dulbecco’s modified eagle medium (DMEM) medium was purchased from HyClone (Marlborough, MA, USA); fetal bovine serum (FBS) were purchased from Biological Industries (Beit Haemek, Israel); nitric oxide fluorometric assay kit was purchased from Beyotime Biotechnology (Shanghai, China).

#### 3.4.2. Cell Culture 

RAW 264.7 macrophages (IBMS, CAMS/PUMC, Beijing China) were maintained in DMEM supplemented with 10% heat-inactivated FBS, 100 U/mL penicillin, and 100 µg/mL streptomycin in a humidified atmosphere containing 5% CO_2_ at 37 °C.

#### 3.4.3. Measurement of NO Levels 

Nitrite, as a major stable product of NO, the level of it measured by Griess reagent was considered to reflect the concentration of NO in culture supernatants. Extract, eluent and compounds obtained from the aerial parts of *P. indica* were used to pretreat the cells for 1 h before stimulating with LPS (500 ng/mL) for 24 h. After incubation, each culture medium (50 µL) was mixed with an equal volume of Griess reagent. An ELISA plate reader was used to determine the nitrite levels at 540 nm, and the concentrations were calculated by referring to a NaNO_2_ standard calibration curve [38].

### 3.5. Statistical Analysis 

Values are expressed as mean ± S.D. SPSS 11.0 was used to conduct the statistics of all the grouped data. *p* < 0.05 was considered to indicate statistical significance. One-way analysis of variance (ANOVA) and Tukey’s Studentized range test were used for the evaluation of the significant differences between means and post hoc, respectively.

## 4. Conclusions

In summary, during the investigation of the chemical compositions from the aerial parts of *P. indica*, twenty-nine compounds, including four new ones, (3′′*R*)-pluthiophenol (**1**), (3′′*R*)-pluthiophenol-4′′-acetate (**2**), 3′′-ethoxy-(3′′*S*)-pluthiophenol (**3**), 3′′-ethoxy-(3′′*S*)-pluthiophenol-4′′-acetate (**4**), along with twenty-five known ones (**5**–**29**) were obtained. The structures of them were determined by means of spectroscopic methods.

Meanwhile, the potential anti-inflammatory effects of compounds **1**–**29** on LPS-stimulated NO production were examined. As a result, compounds **1**, **2**, **10**, **13**, **18**, **23** displayed significant inhibitory activities on LPS-induced NO production at 40 μM, while **3**, **4**, **26**–**29** possessed moderate inhibitory effects. These results suggested that compounds **1**, **2**, **10**, **13**, **18**, **23** may have potent anti-inflammatory activity.

## Figures and Tables

**Table 1:** ^1^H- and ^13^C-NMR data for **1** in CD_3_OD and CDCl_3_.

No.	in CD_3_OD		in CDCl_3_	
	δ_C_	δ_H_ (*J* in Hz)	δ_C_	δ_H_ (*J* in Hz)
2	124.6	—	124.2	—
3	134.9	7.15 (d, 4.0)	133.6	7.10 (d, 4.0)
4	133.3	7.08 (d, 4.0)	132.4	7.04 (d, 4.0)
5	125.9	—	123.8	—
1′	66.8	—	66.4	—
2′	80.1	—	79.6	—
3′	64.6	—	64.1	—
4′	84.5	—	83.6	—
5′	4.2	2.02 (s)	4.8	2.04 (s)
1′′	78.1	—	79.0	—
2′′	94.5	—	91.4	—
3′′	64.6	4.55 (dd, 5.0, 7.0)	63.8	4.68 (dd, 4.0, 6.0)
4′′	66.9	3.64 (dd, 7.0, 11.5)	66.2	3.77 (dd, 6.0, 11.5)
		3.68 (dd, 5.0, 11.5)		3.83 (dd, 4.0, 11.5)

**Table 2:** ^1^H- and ^13^C-NMR data for **2** in CD_3_OD.

No.	δ_C_	δ_H_ (*J* in Hz)	No.	δ_C_	δ_H_ (*J* in Hz)
2	125.0	—	1′′	78.5	—
3	135.0	7.17 (d, 4.0)	2′′	93.3	—
4	133.6	7.10 (d, 4.0)	3′′	61.8	4.76 (dd, 5.0, 6.5)
5	125.5	—	4′′	68.1	4.19 (dd, 6.5, 11.0)
1′	67.0	—			4.21 (dd, 5.0, 11.0)
2′	80.1	—	1′′′	172.5	—
3′	64.7	—	2′′′	20.7	2.08 (s)
4′	84.6	—			
5′	4.1	2.03 (s)			

**Table 3:** ^1^H- and ^13^C-NMR data for **3** in CD_3_OD.

No.	δ_C_	δ_H_ (*J* in Hz)	No.	δ_C_	δ_H_ (*J* in Hz)
2	124.8	—	5′	4.1	2.03 (s)
3	135.0	7.16 (d, 4.0)	1′′	79.5	—
4	133.6	7.10 (d, 4.0)	2′′	92.5	—
5	125.6	—	3′′	72.7	4.34 (t, 5.5)
1′	66.7	—	4′′	65.6	3.69 (d, 5.5)
2′	80.2	—	5′′	66.1	3.55 (dq, 7.0, 9.0)
3′	64.5	—			3.83 (dq, 7.0, 9.0)
4′	84.6	—	6′′	15.5	1.24 (t like, *ca.* 7)

**Table 4:** ^1^H- and ^13^C-NMR data **4** in CD_3_OD.

No.	δ_C_	δ_H_ (*J* in Hz)	No.	δ_C_	δ_H_ (*J* in Hz)
2	125.2	—	1′′	79.9	—
3	135.1	7.17 (d, 4.0)	2′′	91.2	—
4	133.9	7.12 (d, 4.0)	3′′	69.5	4.57 (dd, 4.5, 6.0)
5	125.2	—	4′′	66.5	4.23 (dd, 4.5, 11.5)
1′	66.6	—			4.26 (dd, 6.0, 11.5)
2′	80.3	—	5′′	66.1	3.55 (dq, 7.0, 9.0)
3′	64.5	—			3.81 (dq, 7.0, 9.0)
4′	84.7	—	6′′	15.4	1.24 (t like, *ca.* 7)
5′	4.1	2.03 (s)	1′′′	172.4	—
			2′′′	20.7	2.07 (s)

**Table 5:** Inhibitory effects of positive control, PI, PIE and compounds **1**–**29** obtained from the aerial parts of *P. indica* on NO production in RAW 264.7 macrophages.

No.	NRC (%)	No.	NRC (%)	No.	NRC (%)
Normal	0.6 ± 0.4	**8**	92.6 ± 5.1	**19**	104.8 ± 1.5
Control	100.0 ± 3.1	**9**	101.1 ± 2.2	**20**	95.1 ± 0.6
Dex	62.2 ± 2.6 ***	**10**	77.9 ± 1.5 **	**21**	101.6 ± 2.0
PI	87.8 ± 2.0 **	**11**	100.9 ± 2.8	**22**	103.8 ± 1.9
PIE	77.9 ± 1.2 ***	**12**	94.2 ± 3.9	**23**	52.1 ± 2.3 ***
**1**	84.5 ± 0.9 **	**13**	88.5 ± 1.2 **	**24**	92.5 ± 0.8
**2**	83.4 ± 0.8 **	**14**	101.7 ± 3.2	**25**	93.6 ± 1.2
**3**	86.9 ± 1.9 *	**15**	99.7 ± 2.3	**26**	91.1 ± 0.9 *
**4**	90.1 ± 0.6 *	**16**	101.9 ± 1.4	**27**	90.3 ± 0.8 *
**5**	92.8 ± 0.4	**17**	101.7 ± 0.1	**28**	89.5 ± 0.9 *
**6**	99.6 ± 1.2	**18**	77.6 ± 1.0 ***	**29**	88.7 ± 2.2 *
**7**	103.9 ± 6.7				

Positive control: Dexamethasone (Dex). Nitrite relative concentration (NRC): percentage of control group, which set as 100%. Values represent the mean ± SD of three determinations. * *p* < 0.05; ** *p* < 0.01; *** *p* < 0.001 (Differences between compound-treated group and control group). N = 4. Final concentration was 100 μg/mL for PI and PIE, 40 μM for **1**–**29**, and 1 μg/mL (2.6 μM) for positive control (Dex), respectively.

## References

[1] Panda S.K., Luyten W. (2018). Antiparasitic activity in Asteraceae with special attention to ethnobotanical use by the tribes of Odisha, India. Parasite.

[2] Wang J., Pei Y.H., Lin W.H., Deng Z.W., Qiao L. (2008). Chemical constituents from the stems and leaves of marine mangrove plant *Pluchea indica* (L.) Less. Shenyang Yaoke Daxue Xuebao.

[3] Tan H., Shen Z., Lin W., Yang A., Xing J., Cao Y. (2010). Study on chemical constituents from roots of *Pluchea indica*. Shanghai Zhongyiyao Daxue Xuebao.

[4] Zhou J.S., Wang F.G., Xing F.W. (2010). *Pluchea sagitallis*, a naturalized medical plant in mainland China. Guangxi Zhiwu.

[5] Buapool D., Mongkol N., Chantimal J., Roytrakul S., Srisook E., Srisook K. (2013). Molecular mechanism of anti-inflammatory activity of *Pluchea indica* leaves in macrophages RAW 264.7 and its action in animal models of inflammation. J. Ethnopharm..

[6] Cho J.J., Cho C.L., Kao C.L., Chen C.M., Tseng C.N., Lee Y.Z., Liao L.J., Hong Y.R. (2012). Crude aqueous extracts of *Pluchea indica* (L.) Less. inhibit proliferation and migration of cancer cells through induction of p53-dependent cell death. BMC Complem. Altern. Med..

[7] Noridayu A.R., Hii Y.F., Faridah A., Khozirah S., Lajis N. (2011). Antioxidant and antiacetylcholinesterase activities of *Pluchea indica* Less. Inter. Food Res. J..

[8] Qiu Y.Q., Qi S.H., Zhang S., Tian X.P., Xiao Z.H., Li M.Y., Li Q.X. (2008). Thiophene derivatives from the aerial part of *Pluchea indica*. Heterocycles.

[9] Biswas R., Dutta P.K., Achari B., Bandyopadhyay D., Mishra M., Pramanik K.C., Chatterjee T.K. (2007). Isolation of pure compound R/J/3 from *Pluchea indica* (L.) Less. and its anti-amoebic activities against *Entamoeba histolytica*. Phytomedicine.

[10] Imashiro F., Maeda S., Takegoshi K., Terao T., Saika A. (1983). Hydrogen bonding and conformational effects on carbon-13-NMR chemical shifts of hydroxybenzaldehydes in the solid state. Chem. Phys. Lett..

[11] Sun B.H., Yashikawa M., Chen Y.J., Wu L.J. (2006). Chemistry study of *Stellaria dichatoma* L. var. lanceolata Bge. Shenyang Yaoke Daxue Xuebao.

[12] Qiu Y.Q., Qi S.H., Zhang C., Li Q.X. (2010). Chemical constituents of *Pluchea indica* (II). Zhongcaoyao.

[13] Li B., Zhang Y., Du W.P., Liu H.D., Liu B., Lai X.W., Xu P. (2015). Chemical constituents from shoot of *Phyllostachys edulis* (II). Zhongyaocai.

[14] Chang R.J., Wang C.H., Zeng Q., Guan B., Zhang W.D., Jin H.Z. (2013). Chemical constituents of the stems of *Celastrus rugosus*. Arch. Pharm. Res..

[15] Lee S.J., Jang H.J., Kim Y., Oh H.M., Lee S., Jung K., Kim Y.H., Lee W.S., Lee S.W., Rho M.C. (2016). Inhibitory effects of IL-6-induced STAT3 activation of bioactive compounds derived from *Salvia plebeia* R. Br. Process Biochem..

[16] Baderschneider B., Winterhalter P. (2001). Isolation and characterization of novel benzoates, cinnamates, flavonoids, and lignans from riesling wine and screening for antioxidant activity. J. Agric. Food Chem..

[17] He K., Cao T.W., Wang H.L., Geng C.A., Zhang X.M., Chen J.J. (2015). Chemical constituents of *Swertia kouitchensis* Franch. Zhongguo Zhongyao Zazhi.

[18] Huo L.N., Wang W., Liu Y., Liu X.H., Zhang L., Cheng K., Liu K., Gao H. (2016). Chemical constituents from leaves of *Perilla frutescens* (II). Zhongcaoyao.

[19] Lee T.H., Kuo Y.C., Wang G.J., Kuo Y.H., Chang C.I., Lu C.K., Lee C.K. (2002). Five new phenolics from the roots of *Ficus beecheyana*. J. Nat. Prod..

[20] Jang D.S., Park E.J., Kang Y.H., Vigo J.S., Graham J.G., Cabieses F., Fong H.H.S., Pezzuto J.M., Kinghorn A.D. (2004). Phenolic compounds obtained from stems of *Couepia ulei* with the potential to induce quinone reductase. Arch. Pharm. Res..

[21] Aranya J., Hongjie Z., Ghee T.T., Cuiying M., Nguyen V.H., Nguyen M.C., Nuntavan B.D., Doel S., Harry H.S.F. (2005). Bioactive constituents from roots of *Bursera tonkinensis*. Phytochemistry.

[22] Kwon H.C., Choi S.U., Lee J.O., Bae K.H., Zee O.P., Lee K.R. (1999). Two new lignans from *Lindera obtusiloba* Blume. Arch. Pharm. Res..

[23] Xiong L., Zhu C., Li Y., Tian Y., Lin S., Yuan S., Hu J., Hou Q., Chen N., Yang Y., Shi J. (2011). Lignans and neolignans from *Sinocalamus affinis* and their absolute configurations. J. Nat. Prod..

[24] Lin S., Chen T., Liu X.H., Shen Y.H., Li H.L., Shan L., Liu R.H., Xu X.K., Zhang W.D., Wang H. (2010). Iridoids and lignans from *Valeriana jatamansi*. J. Nat. Prod..

[25] Yang Y.N., Huang X.Y., Feng Z.M., Jiang J.S., Zhang P.C. (2014). Hepatoprotective activity of twelve novel 7′-hydroxy lignan glucosides from *Arctii Fructus*. J. Agric. Food Chem..

[26] Lee J., Lee D., Jang D.S., Nam J.W., Kim J.P., Park K.H., Yang M.S., Seo E.K. (2007). Two new stereoisomers of tetrahydrofuranoid lignans from the flower buds of *Magnolia fargesii*. Chem. Pharm. Bull..

[27] Heymann H., Tezuka Y., Kikuchi T., Supriyatna S. (1994). Constituents of *Sindora sumatrana* Miq. I. Isolation and NMR spectral analysis of sesquiterpenes from the dried pods. Chem. Pharm. Bull..

[28] Ascari J., Boaventura M.A.D., Takahashi J.A., Duran-Patron R., Hernandez-Galan R., Macias-Sanchez A.J., Collado I.G. (2011). Biotransformation of bioactive isocaryolanes by *Botrytis cinerea*. J. Nat. Prod..

[29] Du C.F., Tu P.F., Chang H.T. (2006). Studies on the chemical references of *Cortex dietamni*. Drug Stand. Chin..

[30] Somsak N., Peerawit P., Chusri T. (2015). Hypoglycemic activity in diabetic rats of stigmasterol and sitosterol-3-*O*-β-d-glucopyranoside isolated from *Pseuderanthemum palatiferum* (Nees) Radlk. leaf extract. J. Med. Plants Res..

[31] Marc von C., Michael A.R.M. (2017). Catalytic oxyfunctionalization of methyl 10-undecenoate for the synthesis of step-growth polymers. Macromol. Chem. Phys..

[32] Holecek J., Lycka A., Micak D., Nagy L., Vanko G., Brus J., Raj S.S.S., Fun H.K., Ng S.W. (1999). Infrared, ^119^Sn, ^13^C and ^1^H-NMR, ^119^Sn and ^13^C-CP/MAS-NMR and moessbauer spectral study of some tributylstannyl citrates and propane-1,2,3-tricarboxylates. Collect. Czech. Chem. Commun..

[33] Shin J.S., Hong Y., Lee H.H., Ryu B., Cho Y.W., Kim N.J., Jang D.S., Lee K.T. (2015). Fulgidic acid isolated from the rhizomes of *Cyperus rotundus* suppresses LPS-induced iNOS, COX-2, TNF-α, and IL-6 expression by AP-1 inactivation in RAW 264.7 macrophages. Biol. Pharm. Bull..

[34] Miura A., Kuwahara S. (2009). A concise synthesis of pinellic acid using a cross-metathesis approach. Tetrahedron.

[35] Chen X.M., Zhou W.W., Wang C.L., Guo S.X. (2014). Study on chemical constituents of mycelium of *Polyporus umbellatus* (pers.) Fries. Zhongguo Xiandai Zhongyao.

[36] Guerrero-Vasquez G.A., Galarza F.A.D., Molinillo J.M.G., Andrade C.K.Z., Macias F.A. (2016). Enantioselective total syntheses of (*R*)- and (*S*)-naphthotectone, and stereochemical assignment of the natural product. Eur. J. Org. Chem..

[37] Bitew H., Mammo W., Hymete A., Yeshak M.Y. (2017). Antimalarial activity of acetylenic thiophenes from *Echinops hoehnelii* Schweinf. Molecules.

[38] Zhang Y., Chao L., Ruan J., Zheng C., Yu H., Qu L., Han L., Wang T. (2016). Bioactive constituents from the rhizomes of *Dioscorea septemloba* Thunb. Fitoterapia.

